# HER3 and LINC00052 interplay promotes tumor growth in breast cancer

**DOI:** 10.18632/oncotarget.14313

**Published:** 2016-12-27

**Authors:** Ahmad Salameh, Xuejun Fan, Byung-Kwon Choi, Shu Zhang, Ningyan Zhang, Zhiqiang An

**Affiliations:** ^1^ Texas Therapeutics Institute, Brown Foundation Institute of Molecular Medicine, The University of Texas Health Science Center at Houston, Houston, Texas, USA; ^2^ Department of Molecular and Human Genetics Virology and Microbiology, Baylor College of Medicine, Houston, Texas, USA; ^3^ Clinical Research Center, First Affiliated Hospital of Nanjing Medical University, Nanjing, Jiangsu, China

**Keywords:** lncRNA, MCF7, T47D, HER3, NRG-1

## Abstract

Here we report that the lncRNA *LINC00052* expression correlates positively with HER3/ErbB3 levels in breast cancer cells. Gene silencing of *LINC00052* diminished both *LINC00052* and HER3 expression and reduced cancer cell growth *in vitro* and *in vivo*. *LINC00052* overexpression promoted cancer cell growth *in vitro* and *in vivo* and increased HER3-mediated downstream signaling. Importantly, neutralization of HER3 signaling with HER3 targeting monoclonal antibodies blocked *LINC00052* mediated cancer cell proliferation *in vitro* and tumor growth *in vivo*, suggesting *LINC00052* promoting cancer growth through HER3 signaling. Taken together, our results indicate that high *LINC00052* levels predict activation of HER3-mediated signaling, and *LINC00052* expression level may serve as a potential biomarker for HER3 targeted antibody cancer therapies.

## INTRODUCTION

Studies have implicated lncRNAs in the etiology of a diverse array of cancer types, asserting that lncRNAs have essential roles in tumorigenesis and occupy a critical space in cancer progression and metastasis [1–4]. LncRNA studies have provided us with new perspective for understanding mechanisms of cancer [5, 6]. LncRNAs furnish potential biomarkers for diagnosis and targeting therapies for cancer [7–12]. For example, the lncRNA *prostate cancer antigen 3* (*PCA3_DD3_*) is a well-documented specific biomarker for prostate cancer [13]. *PCA3* regulates a tumor suppressor, PRUNE2 (a human homolog of the *Drosophila* prune gene), via adenosine deaminase and acting on RNA (ADAR)-dependent adenosine-to-inosine RNA editing [6].

HER3/ErbB3 is a member of the EGFR family of tyrosine kinase receptors (TKRs), which plays a critical role in normal cell growth and development. Upregulation of HER3 has been implicated in the development and progression of various types of cancer [14, 15]. Upon stimulation by the ligand neuregulin (NRG), HER3 heterodimerizes with other members of the EGFR family, which results in its C-terminal tyrosine-phosphorylation and activation of signaling [14, 16]. HER3 activation is associated with resistance to several targeted cancer therapeutics including those targeting HER2 and EGFR [17, 18]. Despite the strong evidence regarding the role of HER3 in cancer, current understanding of the regulation of HER3 expression and signaling in cancer is still limited [14]. The lack of established biomarkers for identification of HER3 driven cancer poses a big challenge in the clinical development of HER3 targeting antibodies [14].

A recent report revealed involvement of lncRNAs in HER2-enriched subtype breast cancer [4]. However, there is no report on lncRNAs in relation to HER3 in the context of cancer. In this study, we report the interplay of the lncRNA *LINC00052* and HER3, and the implication of the lncRNA *LINC00052*/HER3 expression in breast cancer development. Our results from both *in vitro* cell-based studies and *in vivo* animal models indicate that *LINC00052* expression level represents a potential new biomarker for HER3-targeting cancer therapies.

## RESULTS

### *LINC00052 correlates with* HER3 expression in cancer cells

**Figure 1:**
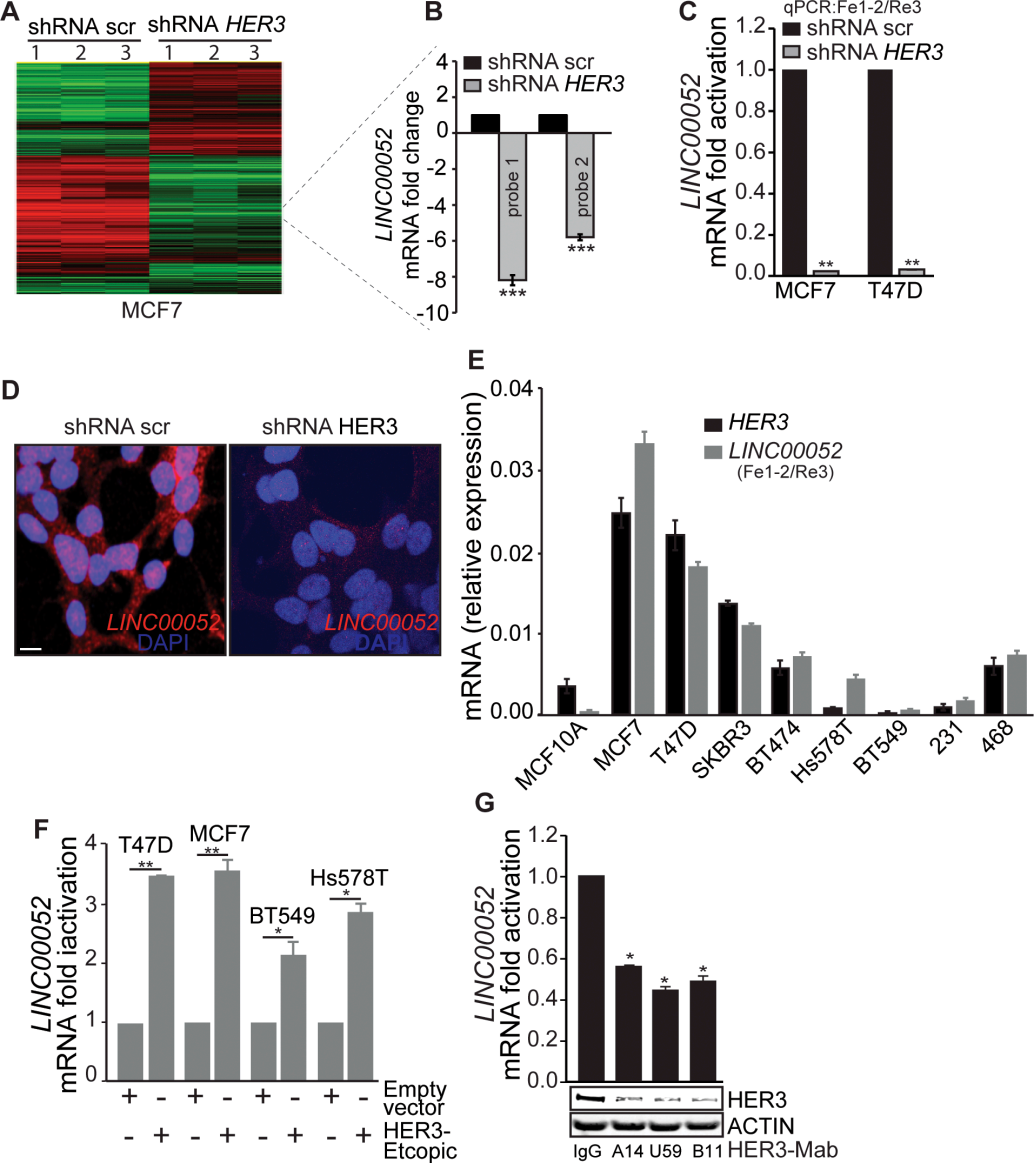
LINC00052 level correlates HER3 expression in breast cancer cells (**A**) Heatmap showing the clustering of gene expression for three independent microarrays (referred as 1, 2 and 3) for MCF7/HER3-knockdown and scramble (control-shRNA) cells. (**B**) Two independent probes for *LINC00052* display significant (***p-value < 0.0001) down-regulation of *LINC00052* in MCF7/HER3-knockdown cells compared to scramble-shRNA (control) cells. (**C**) qRT-PCR analysis (using Fe-1-2 and Re3 primers, locations are indicated in the Supplementary Figure S1A and Supplementary Table S1) for *LINC00052* expression in MCF7 and T47D stably expressing shRNA HER3 or control constructs (shRNA scr). (**D**) RNA-fluorescence *in situ* hybridization (FISH) of *LINC00052* in MCF7 cells expressing scramble or HER3-shRNA constructs. Expression is noted in both nucleus and cytoplasm, bar = 10 μm. (**E**) Breast cancer cells (MCF10A, MCF7, T47D, SKBR3, BT474, Hs578T, BT549, MDA-MB-231 and MDA-MB-468), grown to reach 80% confluence in 10% fetal serum bovine (FBS), were assessed for *LINC00052* and HER3 RNA by qRT-PCR (reported as relative expression). (**F**) Levels of *LINC00052*, normalized *GAPDH*, assessed by qRT-PCR, in breast cancer cells (T47D, MCF7, BT549, and Hs578T) stably expressing ectopic HER3. (**G**) Evaluation of *LINC00052*, HER3, and ACTIN expression in MCF7 cells subjected for 24 hrs of treatment with 10 μg HER3 blocking antibodies (HER3-Mabs: A14, U59, and B11) or IgG isotype control. In each case, shown is mean ± S.D. of a representative of triplicate experiments *p < 0.05, **p < 0.001 and ***p < 0.0001 (Student's *t*-test, knockdown or ectopic-HER3 vs control cells).

While HER3 expression and signaling are well studied [16, 19, 20], the role of HER3 signaling in transcriptional regulation remains largely unknown. Using a DNA-microarray, we analyzed gene expression profiles in MCF7 cancer cells (an epithelial-luminal breast cancer cell line) stably transduced with *HER3*-shRNA for HER3 silencing in comparison with scramble-shRNA control cells. The gene profiling analysis identified a number of genes with significant changes in expression level in response to HER3 knockdown, including the lncRNA *LINC00052* (Figure 1A and 1B). *LINC00052*, with previously unknown function, is significantly reduced in HER3-knockdown MCF7 breast cancer cells in comparison with the control cells as indicated by two independent probes (8.1 and 5.8 fold change of mRNA levels for probe 1 and probe 2, respectively, *p* ≤ 0.0001) (Figure 1B). Genomic *in silico* analysis shows *LINC00052* as a lncRNA (ENSG00000259527) that generates a single predicted primary transcript of 2.94 kb with a mature RNA of 1.966 kb. *LINC00052* is located on the positive (+) strand and encompasses the region chr15:87,576,929–87,579,866 (Supplementary Figure S1A). Genome comparative analysis showed that the 3′-end of *LINC00052* is highly conserved among mammals (Supplementary Figure S1B) and high homology was found in primate species-conserved tracks (Supplementary Figure S1B and S1C) suggesting a conserved functional role. Although lncRNAs are rarely translated, studies suggest that a class of bifunctional RNAs encoding both mRNAs and functional noncoding transcripts may exist [21–23]. We examined the *LINC00052* DNA sequence for potential translational initiation and termination codons and performed immunoblotting analysis. Our data showed no detectable protein product of *LINC00052* bearing FLAG-tag inserted before the potential stop-codon (Supplementary Figure S1D).

To confirm the results from the gene profiling study, we evaluated *LINC00052* expression using quantitative PCR (qPCR) in both MCF7 and T47D breast cancer cell lines stably transduced with *HER3*-shRNA (Supplementary Figure S2A–S2D) in comparison with the corresponding scramble control constructs. Levels of endogenous *LINC00052*, pre-mRNA and mRNA decreased with *HER3* knockdown (Figure 1C; Supplementary Figure S2A–S2D). We further confirmed these findings by FISH analysis where *HER3* knockdown also resulted in a reduced endogenous *LINC00052* expression in both cytoplasm and nucleus in comparison with the shRNA scramble control (Figure 1D).

Next, we evaluated *LINC00052* expression in a panel of breast cancer cell lines with different levels of HER3 expression. Consistently, *LINC00052* expression showed positive correlation with HER3 in human breast cancer cells. Cancer cells (MCF7, T47D, and SKBR3) with relatively high-HER3 expression showed higher *LINC00052* levels, while low-HER3 expressing cancer cells such as BT549 and MDA-MB-231 showed low *LINC00052* levels (Figure 1E). These results indicate a tight correlation between *LINC00052* and HER3 expression in breast cancer cells. To further confirm the correlation between HER3 and *LINC00052* expression, we established breast cancer cells stably expressing ectopic-HER3. Quantitative RT-PCR analysis showed upregulation of *LINC00052* in cancer cells ectopically expressing HER3 when compared with the empty vector control cells (Figure 1F). Furthermore, we treated cancer cells with a panel of anti-HER3 monoclonal antibodies (HER3-Mab) and inhibition of HER3 by the neutralizing antibodies (referred as A14, U59, and B11) resulted in significant decreasing of *LINC00052* expression when compared to an IgG isotype control (Figure 1G). Collectively, our data indicate that *LINC00052* expression is positively correlated with HER3 levels.

### Phosphorylated HER3 correlates with *LINC00052* expression

**Figure 2:**
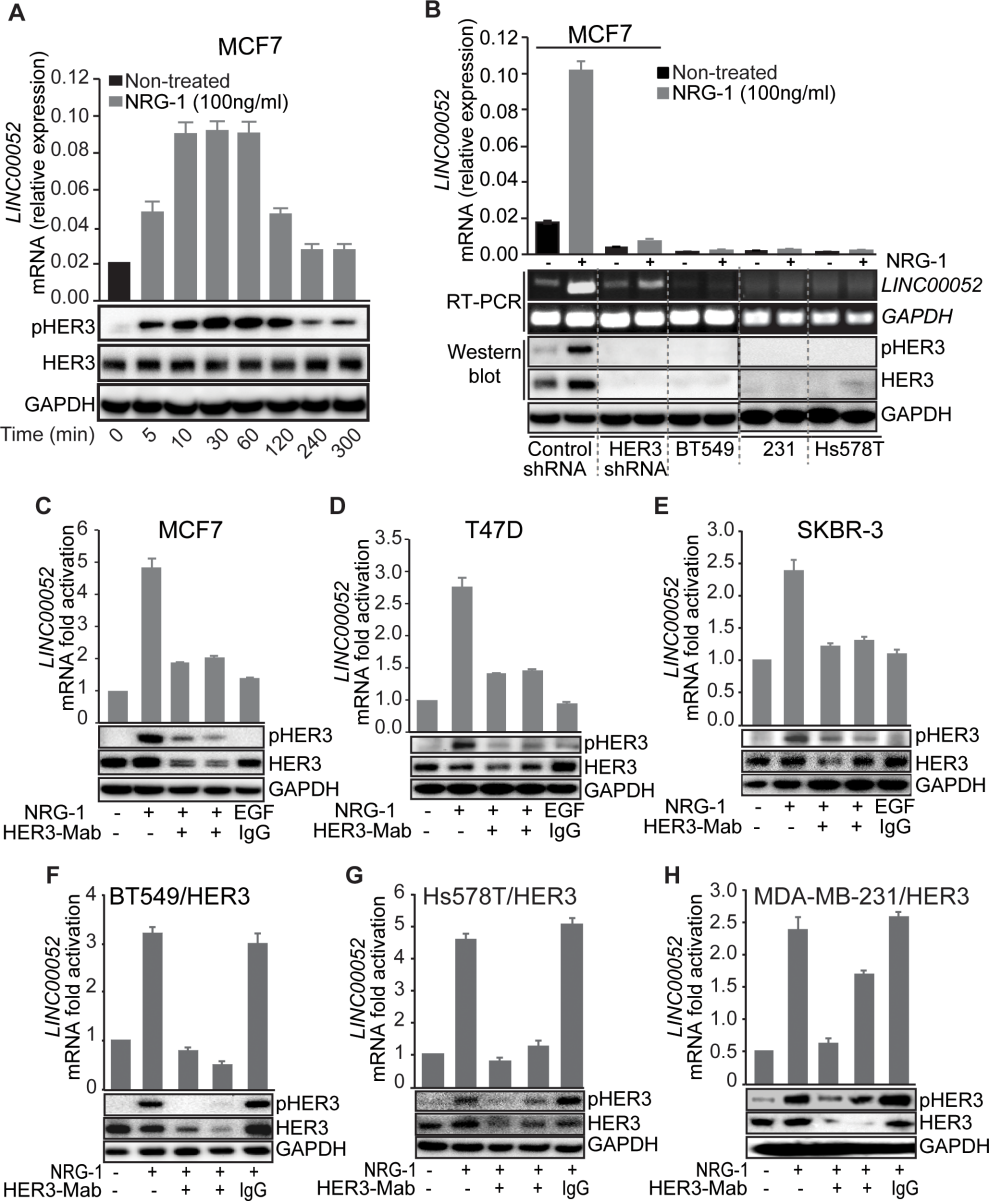
HER3 activation induces LINC00052 expression in breast cancer cells (**A**) MCF7 cells were serum-starved, treated with 100 ng/ml NRG-1 (in time-course intervals) or vehicle and evaluated for *LINC00052*, pHER3, HER3, and GAPDH levels by qRT-PCR and immunoblotting analysis. (**B**) Breast cancer cell MCF7/shRNA-scramble, MCF7/shRNA-HER3, BT549, MDA-MB-231 and Hs578T) were stimulated with 100 ng/ml NRG-1 or vehicle and subjected to Western blot or qRT-PCR for the evaluation of *LINC00052*, HER3, pHER3, or GAPDH levels. (**C**–**E**) Breast cancer cells MCF7, T47D, and SKBR3 were grown in 10% fetal serum bovine to reach 85% confluence followed by 18 hrs serum-starvation. Subsequently cells were pre-treated for 30 min with 10 μg/ml of HER3 blocking monoclonal antibodies (HER3-Mabs: B14 or U59), followed by 60 min stimulation with 100 ng/ml of NRG-1 (+) or untreated (−) as indicated. EGF-treatment was used as negative control. *LINC00052* and HER3 RNA relative expression were analyzed by qRT-PCR and whole cell lysates were analyzed by Western blot with antibodies against HER3, pHER3 and GAPDH. (**F**–**H**) Breast cancer cells that have low expression of HER3 (BT549, Hs578T and MDA-MB-231) stably expressing ectopic-HER3 were serum-starved and pre-treated for 30 min with HER3-targeting antibodies B14 or U59 or IgG isotype control followed by 60 min stimulation with 100 ng/ml NRG-1. *LINC00052* and *HER3* RNA relative expression were analyzed by qRT-PCR. Whole cell lysates were analyzed by Western blot with antibodies against HER3, pHER3, and GAPDH. In each case, shown is mean ± S.D. of a representative of triplicate experiments.

After establishing the correlation of *LINC00052* expression with HER3 levels in breast cancer cells, we then investigated the relationship between *LINC00052* expression and HER3 phosphorylation. A time course study of *LINC00052* expression upon HER3 activation by NRG-1 stimulation was performed. We observed that, upon stimulation with NRG-1, MCF7 breast cancer cells displayed a robust increase of *LINC00052* RNA levels along with increased HER3 phosphorylation (Figure 2A). *LINC00052* RNA level began to decrease at 120 minutes upon NRG-1 stimulation, which coincides with the decrease of HER3 phosphorylation (Figure 2A). Further, we examined *LINC00052* expression in breast cancer cells of HER3-high (MCF7), HER3-knockdown (MCF7/HER3 KD) or HER3-low (BT549, MDA-MB-231 and Hs578T) upon stimulation with NRG-1, and results showed that *LINC00052* expression was increased in cells with HER3-high but not knockdown- or HER3-low (Figure 2B). These results indicate that *LINC00052* expression is correlated with HER3 activation upon NRG-1-stmulation. Consistently, neutralizing monoclonal antibodies inhibited HER3 activation and resulted in a decrease of *LINC00052* levels concomitantly with reduction of total HER3 and pHER3 in comparison to controls (Figure 2C–2E). To determine if the change of *LINC00052* expression is HER3 specific, we treated cancer cells with lapatinib (a compound that inhibits kinase activity of HER2 and EGFR) or trastuzumab (a HER2-blocking antibody). Results showed no significant change of *LINC00052* levels upon treatment with lapatinib or trastuzumab in comparison to the vehicle control (Supplementary Figure S3A–S3E). To further validate these findings, we expressed HER3 ectopically in breast cancer cells with low endogenous HER3 (BT-549, MAD-MB-231 and Hs578T). Consistently, qRT-PCR results showed increased *LINC00052* upon stimulation by NRG-1 when cells had ectopically expressed HER3, and anti-HER3 antibodies can inhibit the levels of *LINC00052* (Figure 2F–2H). Collectively, these results indicate that expression of the long noncoding RNA *LINC00052* is correlated with HER3 phosphorylation.

### *LINC00052* induces HER3 expression in breast cancer cells

**Figure 3:**
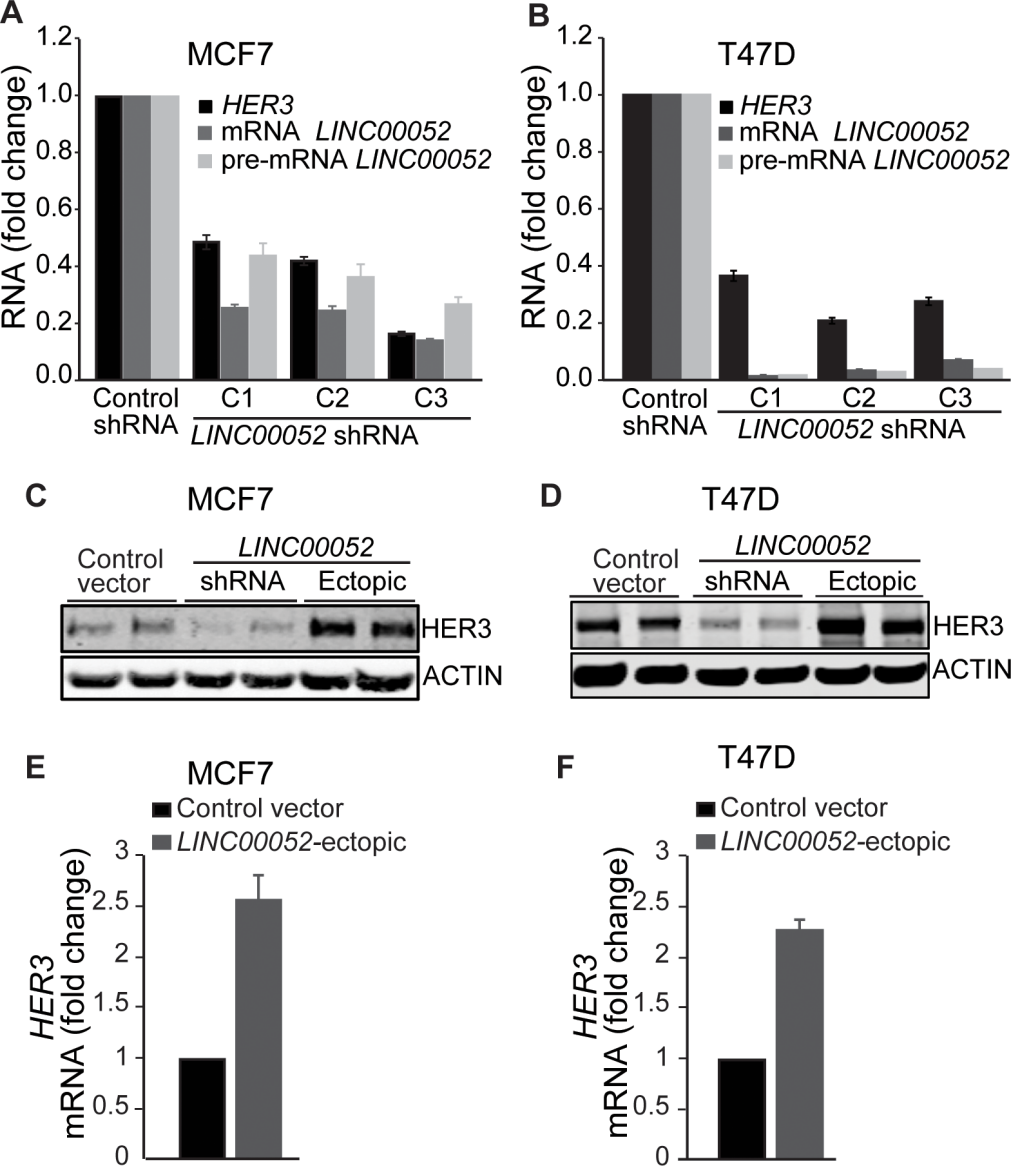
LINC00052 increases HER3 expression in breast cancer cells (**A**, **B**) Breast cancer cells MCF7 or T47D stably expressing three different lentiviral *LINC00052*-silenced shRNA or shRNA-scramble (control) constructs were grown in 10% fetal serum bovine to reach 85% confluence and subsequently assessed for HER3 and *LINC00052* expression by qRT–PCR. (**C**, **D**) Whole cell lysates from breast cancer cells MCF7 or T47D stably expressing shRNA-control, GFP, *LINC00052* shRNA-silenced or *LINC00052*-ectopic lentiviral constructs were analyzed by immunoblotting with antibodies against HER3 or ACTIN. (**E**, **F**) Evaluation of *HER3* RNA levels by qRT-PCR in breast cancer cells MCF7 and T47D stably expressing *LINC00052*-ectopic or control constructs.

Studies have shown that lncRNAs can function in a regulatory feedback-loop through cis- or trans-acting genes [6, 24, 25]. We sought to investigate a possible role of *LINC00052* in the regulation of HER3 expression in breast cancer cells. To test this possibility, we altered *LINC00052* expression in breast cancer cells MCF7 and T47D by knockdown of *LINC00052* using shRNA-silencing or ectopically expressing full non-spliced RNA *LINC00052*. Results revealed decreasing levels of HER3 expression upon *LINC00052*-knockdown in comparison to control cells (Figure 3A and 3B; Supplementary Figure S4A and S4B). In contrast, we observed increasing levels of HER3 protein and RNA when *LINC00052* was ectopically overexpressed in comparison to control cells (Figure 3C–3F; Supplementary Figure S4C and S4D). To further demonstrate the functional role of *LINC00052* in regulating HER3, we performed additional experiments evaluating the other HER family members in breast cancer cells stably expressing the full non-spliced *LINC00052* RNA. Ectopic overexpression of *LINC00052* induced upregulation of HER3 but not of *EGFR*, *HER2* or *HER4* mRNA (Figure 3E and 3F; Supplementary Figure S4E and S4F). Taken together, these data indicate that *LINC00052* specifically increases HER3 expression in breast cancer cells.

### *LINC00052* promotes cancer cell growth by increasing HER3 signaling

**Figure 4:**
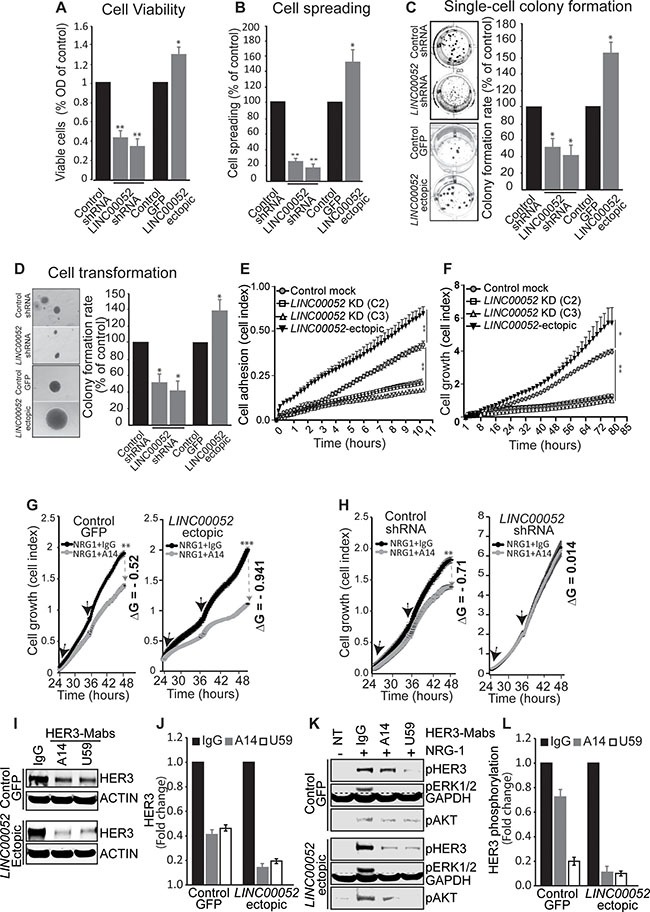
LINC00052 functions in breast cancer cells (**A**–**F**) MCF7 breast cancer cells stably expressing *LINC00052*-shRNA, *LINC00052*-ectopic or control lentiviral constructs were subjected to functional assays. (A) MCF7 cell were seeded at 50% confluence in complete medium and grown for 72 hours, cell viability was evaluated using the AlamarBlue assay. Quantitative histograms (fold change) of cell growth rate compared to control cells are shown (representative cell images are reported in Supplementary Figure S5A). (B) MCF7 spheroids harvested after 16 hrs and plated in 12-well plates for 24 hrs. Representative images from 6 spheroids (for each cell line) were evaluated for spreading cell activity (cells migrated out of the spheroid “borders” {arbitrary black linet) were counted and reported as % of spreading of control cells (representative cell images are shown in Supplementary Figure S5B). (C) Single-cell colony formation assay, transduced MCF7 cells (as indicated) were plated in 12-well plates at extremely low density (10–20 cells/cm^2^) and grown in RPMI medium supplemented with 10% FBS for 4 weeks. Colony number and areas were measured by ImageJ and reported as % of control cells. Representative images and colony formation rate percentage (%) of control cells are reported (additional representative images are shown in Supplementary Figure S5C). (D) Colony formation assay in soft agar. Colonies of transduced MCF7 cells were cultured in 0.35% soft agar in RPMI medium supplemented with 10% FBS. Cells were assessed for anchorage-independent growth for 4 weeks. Representative images (from optical miscroscopy) of single colonies are reported, quantification of ColonyArea are roprted as rate percentage (%) of control cells (additional representative images are shown in Supplementary Figure S5D). (E, F) Effect of *LINC00052* knockdown or its ectopic expression on cell adhesion (E) and growth (F). Cells were grown in a pre-coated-fibronectin 96-well E-Plate (ACEA Biosciences) in the presence of RPMI containing 10% (vol/vol) FBS plus NRG-1 and monitored over time by an xCELLigence Real-Time Cell Analysis (RTCA) system for adhesion (over 11 hrs) and growth (over 85 hrs). (**G**, **H**) Effect of HER3-neutralizing antibody (A14) on the proliferation of MCF7 breast cancer cells stably-transduced for *LINC00052*-ectopic, control-GFP lentiviral constructs, *LINC00052*-shRNA or control shRNA. Serum-starved cells were grown for 24 hrs in RPMI-medium supplemented with 0.5% FBS plus 100 ng/ml NRG-1. Subsequently, cells were treated with HER3-Mab A14 (10 mg/ml refreshed every 12 hrs, as indicated with the black arrows. Cell growth (reported as cell index) was monitored for over time (one read every 15 min) by an xCELLigence Real-Time Cell Analysis (RTCA) system. Change of growth (ΔG) was calculated as: ΔG = (Growth with IgG – Growth with HER3-Mab). (**I**, **J**) Evaluation of HER3 protein levels by Western blot in whole cell extracts derived from *LINC00052*-ectopic or control cells subjected to HER3-Mabs (A14, U59) or IgG treatment for 24 hours. (**K**, **L**) Immunoblotting analysis of whole cell extracts derived from *LINC00052*-ectopic or control cells subjected to treatment with HER3-Mabs or isotype IgG (control) for 24 hrs followed by treatment with NRG-1 or vehicle (NT) for 30 min. In each experiment, mean ± SD is shown. *p < 0.05; **p < 0.01 and ***p < 0.0001 (Student's *t*-test).

Recent studies showed that some lncRNAs have essential roles in tumorigenesis, cancer progression and metastasis [1–4]. To understand whether *LINC00052* expression promotes growth of breast cancer cells in a high HER3 context, we investigated the function of *LINC00052* in MCF7 breast cancer cells stably expressing *LINC00052*-silenced shRNA, ectopic *LINC00052*, in comparison with controls. Results showed that elevation of *LINC00052* expression in MCF7 cells resulted in enhanced cell survival, spheroid-spreading capability, single cell colony formation, anchorage-independent growth, adherence, and cell growth (Figure 4A–4F; Supplementary Figure S5A–S5D). MCF7 cells with *LINC00052*-shRNA displayed decreased biological activities measured in these assays as compared to controls (Figure 4A–4F; Supplementary Figure S5A–S5D). These results showed that increase of *LINC00052* expression promotes not only cell growth and survival, but also cell adhesion, spreading, and transformation of breast cancer cells. Next, we sought to test whether HER3-neutralizing antibodies could block tumor promoting activity of *LINC00052* in breast cancer cells stably expressing ectopic-*LINC00052*. Indeed, results showed that MCF7 cells with ectopic expression of *LINC00052* were more responsive to HER3 blocking antibody treatment. MCF7 cells ectopic expressing *LINC00052* expression (*LINC00052*-ectopic) showed significant inhibition of cell growth by a HER3 blocking antibody as compared with control cells (Figure 4G). Furthermre, we observed that *LINC00052*-ectopic fails to increase the cell growth of breast cancer MCF7 when HER3 is silenced by shRNA, suggesting that *LINC00052* promotes cell growth by increasing HER3 expression (Supplementary Figure S5E and S5F). These data indicate that increased *LINC00052* expression renders breast cancer cells more sensitive to HER3-blocking antibodies. Interestingly, MCF7 cells expressing *LINC00052*-silenced shRNA did not show responsiveness to HER3-blocking antibody treatment when compared to control cells (Figure 4H). Of note that reductions of HER3 levels by the neutralizing antibodies against-HER3 were consistently greater in *LINC00052*-ectopic cells than in control cells (Figure 4I and 4J). Further, we evaluated phosphorylation levels of HER3, ERK1/2 and AKT in *LINC00052*-ectopic cells after treatment with HER3-Mabs followed by NRG-1-stimulation. As expected, upon treatment with anti-HER3 antibodies, there was greater reduction of pHER3, pERK1/2, and pAKT in *LINC00052*-ectopic MCF7 cells when compared to control cells (Figure 4K–4L). Collectively, these results demonstrate that *LINC00052* promotes cancer cell growth and survival by upregulating HER3, and that high *LINC00052* levels predict an increased sensitivity of breast cancer cells to HER3 neutralizing antibody treatment.

### *LINC00052* promotes tumor growth in mouse xenograft models

**Figure 5:**
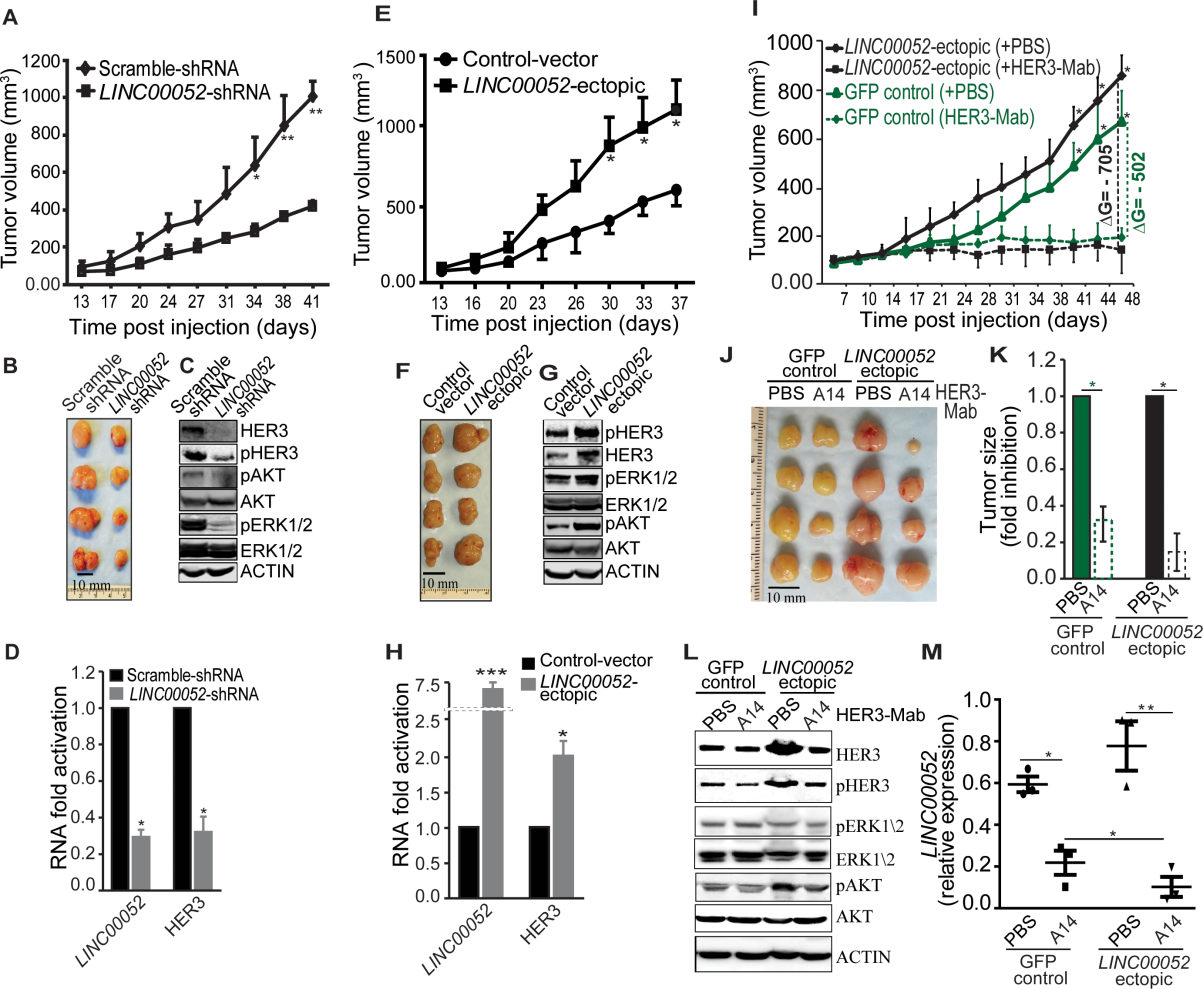
Functions of LINC00052 in breast tumor xenograft models (**A**–**D**) Female immunodeficient nude mice received subcute (sc) injection of 5×10^6^ MCF7 cells stably expressing *LINC00052* silenced or negative control constructs. Tumor volumes, measured twice a week, were plotted over time (A). End-point experiment 6-weeks tumors are shown (B). Immunoblotting analysis of xenograft whole tissue extracts for HER3, pHER3, pAKT, AKT, pERK1/2, ERK1/2 and ACTIN (C). RNA analysis for *LINC00052* and *HER3* levels in the whole tissue extract of xenografts (D). (**E**–**H**) Tumor growth in mice bearing MCF7 xenografts from cells stably expressing *LINC00052*-ectopic or control constructs; volume (mm^3^) plotted over time (E). End-point experiment 6-week tumors are shown (F). Immunoblotting analysis of xenograft whole tissue extracts for HER3, pHER3, pAKT, AKT, pERK1/2, ERK1/2 and ACTIN (G). Quantitative-RT-PCR evaluation of *LINC00052* and *HER3* RNA expression in the whole tissue extract of xenografts (H). (**I**–**M**) *In vivo* HER3-antibody treatment of nude mice bearing MCF7 xenografts of cells stably expressing *LINC00052*-ectopic or control GFP constructs. Two cohorts of SCID mice with size-matched tumors (*n* = 6/group) received 10 μg/gram of mouse weight per dose of HER3-Mab or negative control-PBS. Treated mice and controls received a series of doses (*n* = 9) through intraperitoneal (i.p.) administration twice weekly. Tumor volumes, measured before each administration, were plotted over time (I), and representative tumors at the experimental end-point are shown (J). Evaluation of the change of tumor growth, expressed as ΔG = (average of tumor size treated with HER3-Mab average of tumor size treated with control-PBS) (K). Immunoblotting analysis of xenografts whole tissue extracts for HER3, pHER3, pAKT, AKT, pERK1/2, ERK1/2 and ACTIN (L). Quantitative RT-PCR evaluation of *LINC00052* and *HER3* RNA expression in the whole tissue extract of xenografts (m). In each experiment, 5-6 mice per group were treated. Mean ± SD is shown. *p < 0.05, **P < 0.01.

To directly interrogate the function of *LINC00052* in xenograft tumor models, MCF7 breast cancer cells stably expressing *LINC00052*-shRNA, *LINC00052*-ectopic, or control constructs were subcutaneously implanted in nude mice. Scramble-shRNA cells gave rise to tumors that were, on average, about three times the weight or size of those from mice that received a transplant of cells expressing *LINC00052*-shRNA (Figure 5A and 5B). We performed *ex vivo* immunoblotting and qRT-PCR analysis and detected decreases in levels of HER3, pHER3, pAKT, and pERK1/2 in *LINC00052*-shRNA tumor lysates in comparison with controls (Figure 5C and 5D). In contrast, cancer cells ectopically expressing *LINC00052* showed significant increase of tumor growth when compared to controls (Figure 5E and 5F; Supplementary Figure S6A). Moreover, we detected increased levels of HER3, pHER3, pAKT, pERK1/2 and *LINC00052* in *LINC00052*-ectopic xenograft in comparison to control (Figure 5G and 5H). To explore potential clinical application of these findings, we performed serial administration of an anti-HER3 monoclonal antibody (referred as A14) to tumor-bearing mice with established breast cancer xenografts. We observed significantly higher inhibition of tumor growth in *LINC00052*-ectopic xenograft in comparison with the inhibition of GFP-control xenograft (Figure 5I–5K; Supplementary Figure S6B). Furthermore, we observed decreased levels of HER3, pHER3, pAKT, and pERK1/2 in *LINC00052*-ectopic xenograft treated with HER3 blocking antibody as compared with controls (Figure 5L). Interestingly, qRT-PCR analysis show *LINC00052* levels also decreased after HER3-targeting antibodies treatments (Figure 5M). These results support the notion that *LINC00052* expression plays an important role in HER3 expression and signaling.

## DISCUSSION

Here, we describe for the first time a regulatory interplay between the long noncoding RNA designated as *LINC00052* and HER3 expression in breast cancer cells. Our *in vitro* and *in vivo* data showed that breast cancer cells with high HER3 but not knockdown or low-HER3 levels displayed upregulation of *LINC00052* expression. Interestingly, we found that elevation of *LINC00052* levels induce HER3 expression and signaling but not EGFR, HER2 or HER4 and exerts increased growth of breast cancer cells. In contrast, *LINC00052*-silencing resulted in diminished HER3 expression and cell growth suggesting that *LINC00052* exerts an important role for HER3 regulation in HER3-enriched breast cancer cells. Importantly, our results showed that *LINC00052* expression was strongly suppressed in breast cancer cells subjected to HER3 neutralizing monoclonal antibody treatment. Studies with mouse xenograft model showed that high *LINC00052* levels predict activation of HER3-mediated signaling and may therefore serve as a biomarker for HER3 targeted antibody cancer therapies.

Limited studies have been reported on the role of lncRNAs regulation of the *EGFR* family of tyrosine kinase receptors which includes HER3. A recent study reported the expression of lncRNAs in a HER2-enriched breast cancer subtype [4]. In another report, a cytoplasmic lncRNA regulator of EGFR, LINK-A (long intergenic non-coding RNA for kinase activation), was identified to mediate HB-EGF-triggered, EGFR:GPNMB heterodimer-dependent HIF1α phosphorylation [26]. *LINC00052* has been found expressed in human non-triple negative breast cancer subtypes [27]. Another recent study investigated neurotrophic tyrosine kinase receptor type 3 (NTRK3) as a target gene of *LINC00052*, hypothesizing a role in inhibiting invasion and migration of hepatocarcinoma cells SMMC7721 through complementing with miR- 128 and miR-485-3p and down-regulating NTRK3 [28]. Interestingly, a comprehensive analysis has shown that *LINC00052* is among the lncRNAs marked by more enhancer chromatin modifications (i.e., H3k27ac) in breast cancer MCF7 cells versus the normal cells HMECs [29]. However, additional mechanistic studies of *LINC00052* chromatin modifications will be necessary to investigate the role of its in the regulation of HER3 in breast cancer subtype these modifications might occur. Genomic characterization study of lncRNAs has revealed significant upregulation of numerous lncRNAs in breast cancer (T) versus non-tumor tissue (N) [30] including *LINC00052*. Results show that *LINC00052* is more expressed in tumor versus non-tumor tissues (T/N 6.25, *p* value = 0.00013) [30]. Independently, we performed *in silico* analysis for *LINC00052* and *HER3* expression in the GENT database (http://medical-genome.kribb.re.kr/GENT/). Interestingly, results show that both *LINC00052* and HER3 mRNA expression were elevated in breast cancer compared to normal tissue (Supplementary Figure S7A and S7B). These data support our observation that *LINC00052* plays a role in breast cancer development and progression.

To the best of our knowledge no information has been reported on the regulation of HER3 in relation to lncRNAs expression in the context of normal physiology or cancer. Here, we demonstrated that *LINC00052* expression is correlated with NRG1-HER3 axis and revealed a regulatory interplay between *LINC00052* and HER3 in breast cancer. A large number of studies have showed that expression of lncRNAs is frequently dysregulated in cancer tissues and their expression is strikingly cell type specific as compared with protein-coding genes [30–35]. The relatively high expression of *LINC00052* in breast cancer cells with high HER3 suggests its functional roles in cancer. However, the detailed mechanisms of regulation involving *LINC00052* require further investigation.

Despite the strong evidence regarding the role of HER3 in cancer, a comprehensive mechanism of HER3 regulation of in cancer is incompletely understood. Recently, we identified two novel partners and regulators of HER3. The E3 ubiquitin ligase NEDD4 is a negative regulator of HER3 level and signaling, and NEDD4 expression was inversely correlated with HER3 levels in prostate cancer clinical samples, and its expression was suggested to serve a biomarker for HER3 targeted antibody cancer therapies [20]. DJ-1/PARK7 (Parkinson Protein 7) is a novel interaction partner of HER3 and high DJ-1 expression in breast cancer cells predicts elevated HER3 signaling and may therefore serve as a biomarker for HER3 targeted antibody cancer therapies [36]. The expression and regulation of *LINC00052* could open up interesting new perspectives for the understanding of HER3 regulation in breast cancer. We observed that elevation of *LINC00052*-ectopic expression leads to better response of breast cancer cells to HER3-targeting antibody treatment, suggesting clinical relevance of *LINC00052* as a potential biomarker for HER3-targeting therapies.

## MATERIALS AND METHODS

### Cells, antibodies, and other reagents

Anti-ErbB3 antibody RTJ2 (Abcam); anti-erbB-3/HER-3 antibody, clone 2F12 (Millipore); mouse anti-human c-erbB-3 (BD Biosciences); phospho-HER3/ErbB3 (Tyr1289) (21D3) rabbit mAb, anti-b-actin, anti-b-tubulin (Santa Cruz); anti-GAPDH, anti-AKT, anti-pAKT, anti-pERK1/2, anti-p44/42 MAP kinase (Cell Signaling Technology) were used in the study. Secondary antibodies were purchased from Jackson ImmunoResearch or Invitrogen. DNaseI-RNase free and Ribonucleoside-vanadyl complex were from NEB. Human tumor cell lines used (BT-549, BT-474, Hs587T, MCF-7, MCF10A, SKBR-3, MDA-MB-231, MDA-MB-468 and HEK293) were from ATCC and grown in RPMI or D-MEM containing 10% FBS. The HER3 neutralizing antibodes (HER3Mabs) were produced in our laboratory and described previously [20, 36]. Breefly, the HER3 neutralizing antibodies HER3Mabs are a monoclonal antibodies with backbone of IgG1. HER3Mabs were expressed in HEK293 freestyle cells (Life Technologies) and purified to above 95% purity using protein A/G affinity chromatography. Antibody purity was verified by protein gel electrophoresis and antibody binding was confirmed by ELISA binding assays, flow cytometry analysis. The ability to inhibit HER3 phosphorylation upon NRG-1 activation were verified by ELISA and WBs as described by our group previously [19].

### Microarray

For microarray analyses, we have used three independent preparations of RNA from MCF7 cells stably expressing shRNA-scr (shRNA-scramble) and HER3 (HER3-shRNA lentiviral construct, referred as #619). Total RNA (500 ng) was used for labeling and hybridization according to the manufacturer's protocols (Illumina, Inc) using Illumina's HumanHT-12 v4 Expression BeadChip Kit. The BeadChips were scanned with Illumina BeadArray Reader (Illumina, Inc). The results of microarray data were extracted with Bead Studio 3.7 (Illumina, Inc.) without any normalization or background subtraction. Gene expression data were normalized using quantile normalization method in LIMMA package in R ( www.r-project.org). The expression level of each gene was transformed into a log^2^ before further analysis. To select genes that were differentially expressed in two culture groups, we used a class comparison tool in BRB array tools (v3.6; Biometrics Research Branch, National Cancer Institute, Bethesda, MD) as a method for two-sample *t*-test with the estimation of false discovery rate. To avoid potential false-positive genes because of technical variance, all experiments were carried out in triplicates.

### Bioinformatics and sequence analysis

Chromosomal locations, annotated transcripts, spliced expressed sequence tags, and sequence mapping were visualized on the Genome Browser from the University of California-Santa Cruz ( www.genome.ucsc.edu), by using the latest version of the human genome assembly (hg19) available. Conserved domain analyses were performed through the Conserved Domain Database and sequence alignments were made with either www.ncbi.nlm.nih.gov/Structure/cdd/wrpsb.cgi or http://useast.ensembl.org/index.html.

### Cloning and cDNA generation

Total RNA and genomic DNA from cancer cell lines were isolated through the RNeasy kit (Qiagen) or the All-in-One kit (Norgen Biotek). Total RNA samples from human normal tissues (prostate, brain, liver, kidney, breast, lung, pancreas, spleen, and testis) were commercially obtained (Stratagene). cDNAs were synthesized by using Kit iScript™ cDNA Synthesis (BioRad), SuperScript III reverse transcriptase (Invitrogen or Promega) from total RNA, with N15 random pentadecamers, oligo dT primers, or specific oligonucleotides. Full non-spliced *LINC00052, LINC00052*-FLAG or HER3-FLAG were amplified by PCR with KAPA HiFi DNA polymerase (KAPA Biosystems), cloned under CMV promoter into modified PLVX vector (Clontech) and verified coding sequences were fully sequenced (Supplementary Table S1).

### Small-interfering RNA and short-hairpin RNA

Experiments were performed with retroviral pLKO.1 vectors from the RNAi Consortium (TRC) lentiviral shRNA library (Open Biosystems) expressing specific shRNAs *LINC00052*-silencing: TRCN0000186098 (referred as C1), TRCN0000184966 (referred as C2) and TRCN0000186427 (referred as C3). ShRNAs *HER3*-silencing: TRCN0000040109 (referred as # 40109), TRCN0000000619 (referred as #619) and TRCN0000000623 (referred as # 623). Stable clones were maintained under puromycin selection. Scramble shRNAs or validated non-targeting sequences served as negative controls (Open Biosystems).

### Lentivirus preparation

Lentiviral vectors (pCCLsin.PTT.PGK.EGFP.Wpre, pMDLg/pRRE, pRSV-Rev, and pMD2.VSVG) were used as described [6]. Briefly, HEK293FT cells were transiently transfected (Lipofectamine 2000; Invitrogen) for 16 hours, after which the lentiviruses were harvested 24 and 48 hours later and filtered through 0.22 μm pore cellulose acetate filters. Recombinant lentiviruses were concentrated by Amicon Ultra-15 Centrifugal Filter Units (NMWL of 30 kDa). Lentiviral vector viability was confirmed by reporter gene expression and drug selection.

### Quantitative RT-PCR

RT-qPCR analysis was performed with SYBR-green in a 7500 Fast Real-Time PCR system (Applied Biosystems) or in a Biorad CFX96 Real-Time PCR system. Gene expression levels were normalized against the average of threshold cycle (C_t_) of 3 standard endogenous controls (*GAPDH, ACTIN* or *HPRT*), and the results were analyzed according to the DDCt method. Data were reported as fold induction or relative expression; samples were normalized on to their internal housekeeping genes followed by normalization of each sample to its control. For the oligonucleotides used in this work (Supplementary Table S1).

### RNA fluorescent *in-situ* hybridization and confocal microscopy

To detect *LINC00052* RNA, cells were fixed in 3.6% formaldehyde for 3 min at RT, followed by acetone:methanol 1:1 (vol/vol) for 5 min at 20°C. Cells were permeabilized in PBS containing 0.3% Triton X-100 and 5 mM vanadyl ribonucleoside complex (Invitrogen) on ice for 5 min; vanadyl ribonucleoside complex (an RNase inhibitor) was omitted if the RNase enzymatic activity was to be determined. Cells were washed three times in PBS, for 10 min, and rinsed once in 2× saline-sodium citrate (SSC) buffer prior to hybridization. Hybridization was carried out by using labeled Cy3, DNA-oligonucleotide probes in a moist chamber at 42°C overnight as described [6].

### Cell growth and adhesion assays

Cells were seeded in 200 μl growth medium at a density of 5,000–10,000 cells per well onto E-Plates 96 (ACEA Biosciences). Cell attachment and growth were monitored every 15 min for over 100 hours in the xCELLigence instrument (ACEA Biosciences). Cell growth assays were also performed with the AlamarBlue^®^ cell survival reagent or MTS (Invitrogen, according to manufacturer's instructions), comparable results were observed. For cell adhesion assay, cells were seeded in 200 μl of RPMI medium supplemented with 10% FBS at a density of 5,000–10,000 cells per well onto E-Plates 96. Cell adhesion was monitored every 15 min for 11 hours in the xCELLigence instrument.

### Cell survival assay

Cells were plated at 5–10 × 10^3^ cells per well in 96-well plates and grown for 48 h. The AlamarBlue^®^ dye assay was performed according to the manufacturers protocol. The experiment was performed in quintuplicate. For cell counting, cells were trypsinized and counted using the trypan blue exclusion method to quantify cell viability.

### Analysis of tumor cell-derived spheroids spreading

Tumor cell-derived spheroids were prepared by growing 500–2000 cells in non-adherent 96-microwell culture dishes for 18 hours in RPMI containing 10% FBS and 0.2% methylcellulose. MCF7-derived spheroids were grown for 24 hrs in fibronectin coated Nunc Lab-Tek Chamber Slide system. Representative images of 6 spheroids were evaluated for the number of cells migrated out of the spheroids “borders” (black line, as showed in Supplementary Figure S5B), data are reported as % of spreading of control cells.

### Soft agar colony and single colony formation assay

Cells transduced with the indicated constructs were suspended at either 1500 cells per well in 2 ml of 0.35% low-melting agarose in 6-well plate culture dishes containing 0.7% agarose base. Triplicates were prepared and evaluated for each construct. Colonies were allowed to form at 37°C under standard tissue culture conditions for 4 weeks. After incubation, staining of the colonies with 0.005% crystal violet enabled visual inspection, photographs, and optical density measurements after crystal violet solubilization. For the single-cell colony formation assay, transduced cells (as indicated) were plated in 12-well plates at extremely low density (10–20 cells/cm^2^) and grown in RPMI medium supplemented with 10% FBS for 4–6 weeks. After incubation, staining of the colonies with 0.005% crystal violet enabled visual inspection, photographs, ColonyArea and optical density measurements were performed after crystal violet washing. Representative images and colony formation rate percentage (%) of control cells are reported. Colony number and areas were measured by ImageJ and expressed as % of control cells.

### Western blot analysis

The mixture of cell extract and antibody was then incubated with Pierce™ Protein A/G Magnetic Beads (Thermo Scientific) at 4°C for 2 h, and washed 4 times with NP-40 lysis buffer. The immunoprecipitated proteins were boiled for 5 min in Laemmli sample buffer and separated by SDS–PAGE. Proteins were separated by Proteins were separated by 4–12% bis-Tris NuPAGE (Invitrogen) as indicated, transferred to nitrocellulose membranes, and immunoblotted with specified antibodies.

### RNA and proteins extraction from mouse xenograft of breast tumor tissue

Tumor lysates were prepared as described [20], briefly using the gentleMACS cell dissociator (Miltenyl Biotec) by adding RIPA buffer containing proteinase inhibitor cocktails into finely sliced frozen tumor pieces (1 mg each into 1 mL buffer). RNA and proteins were extracted from 100 l of tissue extracts by using sequential isolation of total RNA, genomic DNA and total proteins from the same sample (Norgen Biotek Corp). Proteins were cleared and quantified by using Pierce™ BCA Protein Assay Kit (Thermo Scientific).

### Tumor-bearing mouse studies

MCF7 (5–10 × 10^6^) cells were injected subcutaneously (s.c.) into each right side of five 6 week-old female nude mice as described [20]. After 10 days tumor volume was measured every 48 h. MCF7 cell lines stably expressing ectopic *LINC00052*, control vector, *LINC00052*-silenced and control-shRNA were used in the studies. In each case, stably expressing pool transduced cells and their corresponding controls were allowed to grow for 48 hours to reach 85% confluence. Afterwards, paired test and control tumor cells were counted, washed in serum-free medium, and re-suspended to a final concentration of 5 × 10^7^/ml in serum- and phenol-free basic RPMI medium. Cells were subsequently mixed in 50% volume of phenol-free Matrigel™ (Becton Dickinson), and cell suspensions (final volume of 200 μl containing 5–10 × 10^6^ cells) were administered subcutaneously in the right flanks of 6-week-old female nude mice. Tumor xenograft growth was monitored serially over time. All animal experimentation was reviewed and approved by the Institutional Animal Care and Use Committee (IACUC) of the University of Texas Health Science Center at Houston.

### Statistical analysis

For the cell proliferation assay of stable cell lines, we performed two-way analysis of variance (ANOVA) at each time point. For other cell line experiments, statistical differences (*P*-values) among groups were obtained using a two-sided Student's *t*-test. All experiments were performed in triplicate. Statistical procedures were performed using Graphpad Prism 5 software (GraphPad Software). We summarized *LINC00052* and *HER3* expression in the samples by the use of standard descriptive statistics for continuous variables or tabulations for categorical variables.

## SUPPLEMENTARY MATERIALS FIGURES AND TABLES



## Abbreviations

AKT: protein kinase B
DAPI: 4′, 6-diamidino-2-phenylindole
EGFR: epidermal growth factor receptor
ERK: extracellular-regulated kinase
FACS: fluorescence-activated cell sorting
FBS: Fetal Bovine Serum
FISH: Fluorescence *in situ* hybridization
HER: human epidermal growth factor receptor
lncRNA: long noncoding RNA
MCF7: an epithelial-luminal breast cancer cell line
NRG: neuregulin
PBS: phosphate buffered saline, shRNA, short hairpin RNA
T47D: an epithelial-luminal breast cancer cell line.
