# Supplementary material for: HER3 and LINC00052 interplay promotes tumor growth in breast cancer
Source: Oncotarget. 2016 Dec 27;8(4):6526–39. doi: 10.18632/oncotarget.14313 (PMC5351650; doi:10.18632/oncotarget.14313)
Supplement: Supplementary file 1 [file oncotarget-08-6526-s001.pdf]

HER3 and *LINC00052* interplay promotes tumor growth in breast cancer

Supplementary Materials

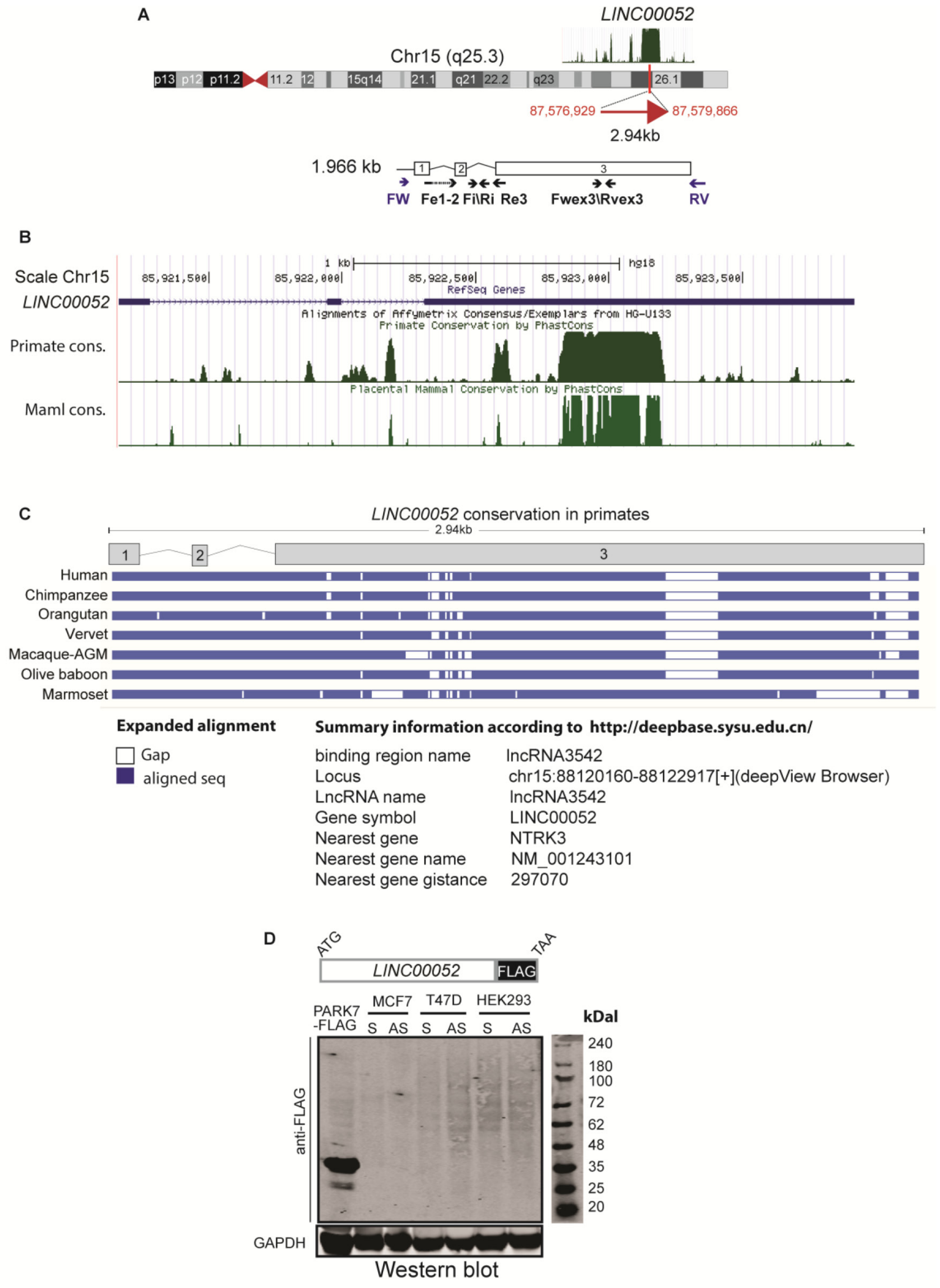

Supplementary Figure S1: Genomic analysis and expression of *LINC00052*. (**A**) Chr15 (q25.3) mapping of *LINC00052* genomic location and its corresponding expected transcriptional unit. The expected pre-mRNA of 2940 bp is marked by the red arrow. Arrows show location of primers used for RT-PCR, qRT-PCR and PCR analysis (FW, RV, Fe-1-2, Re3, Fi/Ri, Fwex3, Rvex3, nucleotide sequences are reported in Supplementary Table S1). (**B**, **C**) Schematic of *LINC00052* conservation across the mammals (B) and primates (C). Genome comparative analysis showed that *LINC00052 3'*-end is highly conserved among mammals and high homology was found in primate species-conserved tracks according to: http://deepbase.sysu.edu.cn/. (**D**) Western blot analysis of potential protein expression of *LINC00052*. cDNA of *LINC00052*-sense (S) and *LINC00052*-antisense (AS) were cloned in frame with FLAG-tag inserted before the predicted stop codon. Resulting constructs were transduced in MCF7, T47D, or 293FT cells. Of note, no trace of *LINC00052* protein product was detected after immunoblotting with antibody against FLAG-tag. Recombinant PARK7 bearing FLAG-tag was used as positive control.

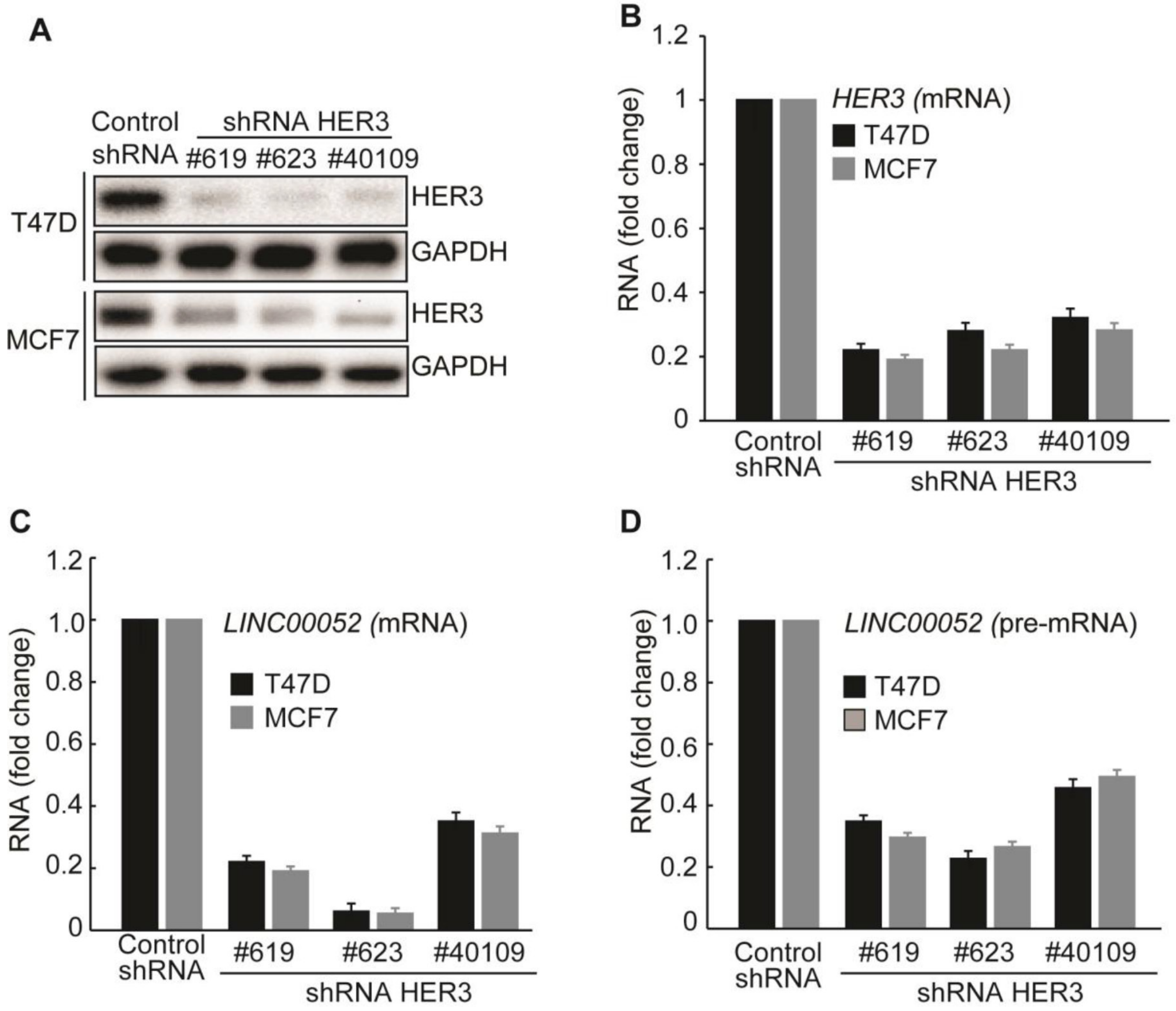

Supplementary Figure S2: HER3-silncing decreases *LINC00052* expression in breast cancer cells. (A–D) Breast cancer MCF7 or T47D cells stably expressing three independent HER3-shRNA or control constructs**.** Western blot analysis for HER3 and GAPDH (A) and evaluation of HER3 mRNA by qPCR (B). Quantitative evaluation of *LINC00052* mRNA (C) and precursor pre-mRNA (D) levels in breast cancer MCF7 or T47D cells stably expressing three independent HER3-shRNA or control constructs.

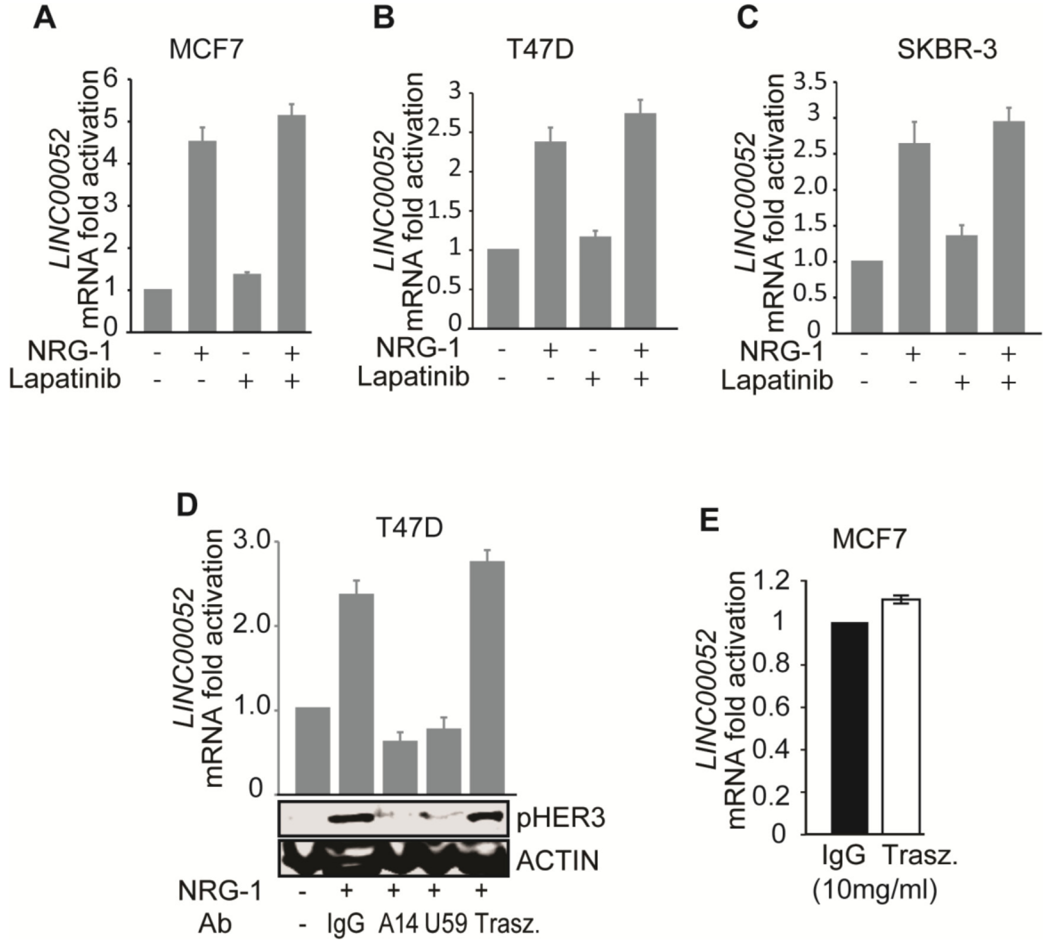

Supplementary Figure S3: Effects of lapatinib or trastuzumab on *LINC00052* expression. (**A–C**) Evaluation of *LINC00052* in breast cancer MCF7, T47D and SKBR-3 cells (grown in 10% FBS) treated with 100 nM of lapatinib or vehicle for 30 min followed by NRG-1-stimulation for 60 min. (**D**) *LINC00052* levels analyzed by qRT-PCR in T47D breast cancer cells treated with HER3 (referred as A14 and U59) and HER2 (Trasz) blocking antibodies. T47D cells were grown in 10% fetal serum bovine to reach 85% confluence followed by 18 hrs serum-starvation. Subsequently, cells were pre-treated for 30 min with 10 µg/ml B14 or U59 (HER3 neutralizing monoclonal antibodies) or *trastuzumab* (HER2 blocking antibody), followed by 60 min stimulation with 100 ng/ml of NRG-1 (+) or untreated (–), as indicated. *LINC00052* and HER3 RNA relative expression were analyzed by qRT-PCR and whole cell lysates were analyzed by Western blot with antibodies against pHER3 and ACTIN. Of note, blocking monoclonal antibodies against HER3 but not against HER2 inhibit *LINC00052* RNA expression upon NRG-1-stimulation. (**E**) Evaluation of *LINC00052* by qRT-PCR in MCF7 breast cancer cells treated with trastuzumab. Cells were grown in 10% fetal serum bovine to reach 85% confluence followed by 18 hrs serum-starvation. Subsequently, cells were pre-treated for 30 min with 10 µg/ml trastuzumab or IgG-isotype (control).

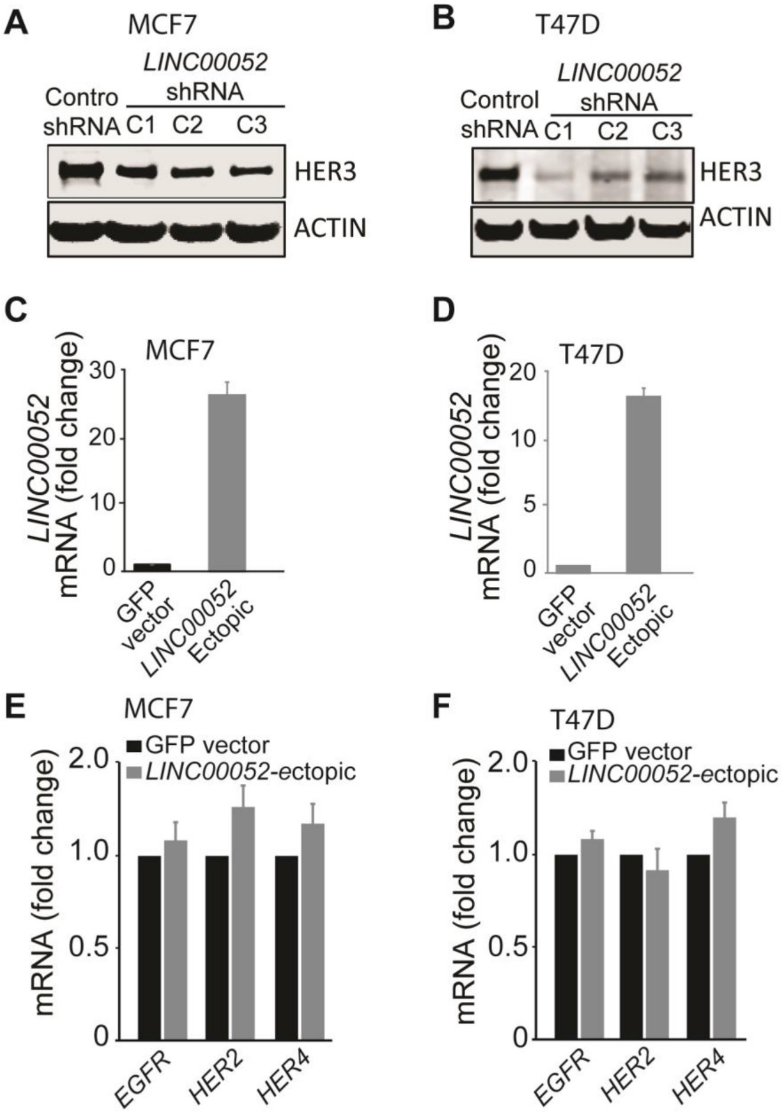

Supplementary Figure S4: Regulation of HER3 expression by *LINC00052.* (**A**, **B**) Breast cancer cells MCF7 or T47D stably expressing three different lentiviral *LINC00052*-silenced shRNA, and control-shRNA constructs were grown in 10% fetal serum bovine to reach 85% confluence and subsequently evaluated for HER3 expression by Western blot. (**C**, **D**) Evaluation of *LINC00052* RNA expression in breast cancer cells MCF7 or T47D stably expressing ectopic *LINC00052* or control constructs (**E**, **F**) Evaluation of *EGFR*, *HER2*, and *HER4* RNA levels (normalized to GAPDH) by qRT-PCR.

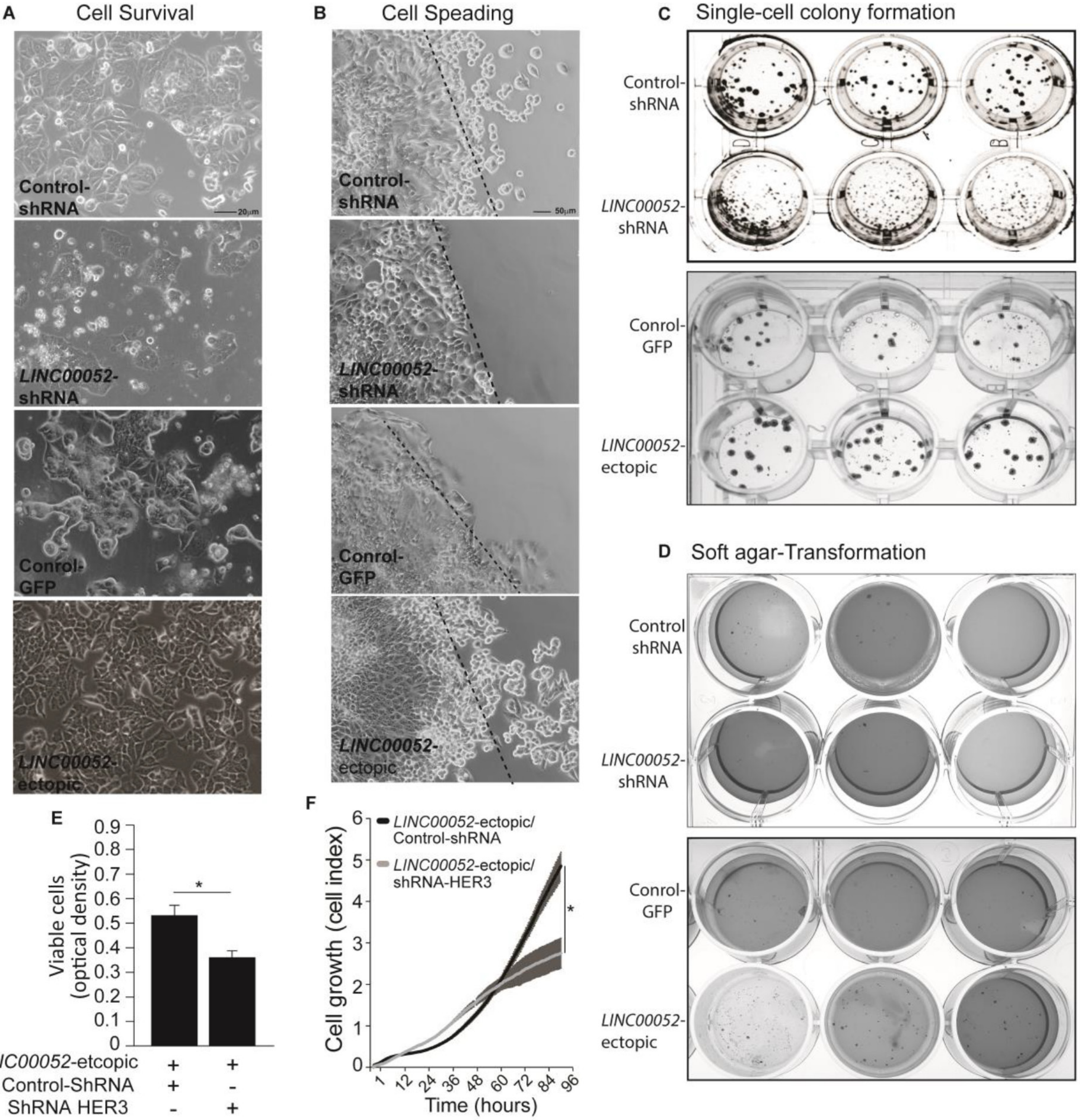

Supplementary Figure S5: *LINC00052* effects in breast cancer cells. (**A**–**D**) MCF7 breast cancer cells stably expressing *LINC00052*-shRNA, *LINC00052*-ectopic or control lentiviral constructs were subjected to functional assays. (A) MCF7 cell were seeded at 50% confluence in complete medium and grown for 72 hrs, representative cell images are shown prior to the cell viability assay. (B) *Spheroids*-spreading, representative images from *spheroids* fixed 24 h after of *spreading* onto *plastic* surface of transduced MCF7 cells (as indicated). Evaluation of spreading activity as number of migrated cells out of the spheroid "borders" (arbitrary black line), counts are reported as % of spreading of control cells. (C) Single-cell colony formation, transduced MCF7 cells (as indicated) were plated in 12-well plates at extremely low density (10 cells /cm^2) and grown in RPMI medium supplemented with 10% FBS for 4 weeks. (D) Transformation activity, transduced MCF7 cells were cultured in 0.35% soft agar in RPMI medium supplemented with 10% FBS. Cells were assessed for anchorage-independent growth for 4 weeks. Representative images of colony formation are reported. (**E**, **F**) Evaluation of cell survival and growth of MCF7, *LINC00052*-ectopic cells transduced with *HER3*-shRNA or control-shRNA constrcuts.

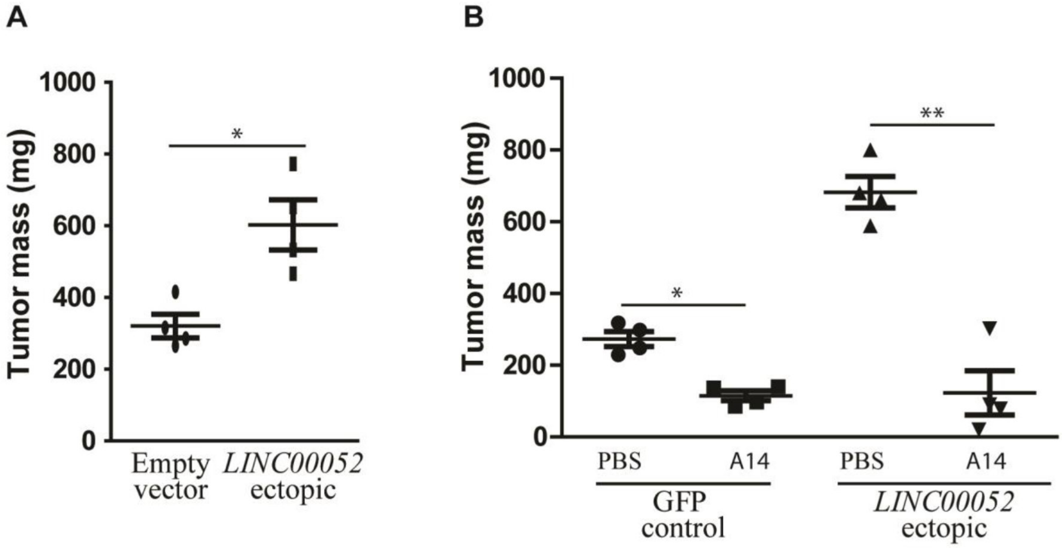

Supplementary Figure S6: Functions of *LINC00052* in MCF7 breast tumor xenograft models. (**A**) Female immunodeficient nude mice received subcutaneous (SC) injection of 5×10^6 MCF7 cells stably expressing *LINC00052* ectopic or scramble control constructs. Experiment end-point tumor mass (mg, milligram) of 6-week tumors are shown. (**B**) *In vivo* HER3 antibody treatment of nude mice bearing MCF7 xenografts of cells stably expressing *LINC00052*-ectopic or control GFP constructs. Two cohorts of SCID mice with size-matched tumors (n = 6/group) received 10 μg/g per dose of HER3 blocking antibody or negative control-PBS. Treated mice received a series of doses (n = 9) through intraperitoneal (i.p.) administration twice weekly. Experimental end-point 6-week tumors mass (mg, milligram) are shown. Mean ± SD is shown. *$p < 0.05$, **$p < 0.01$.

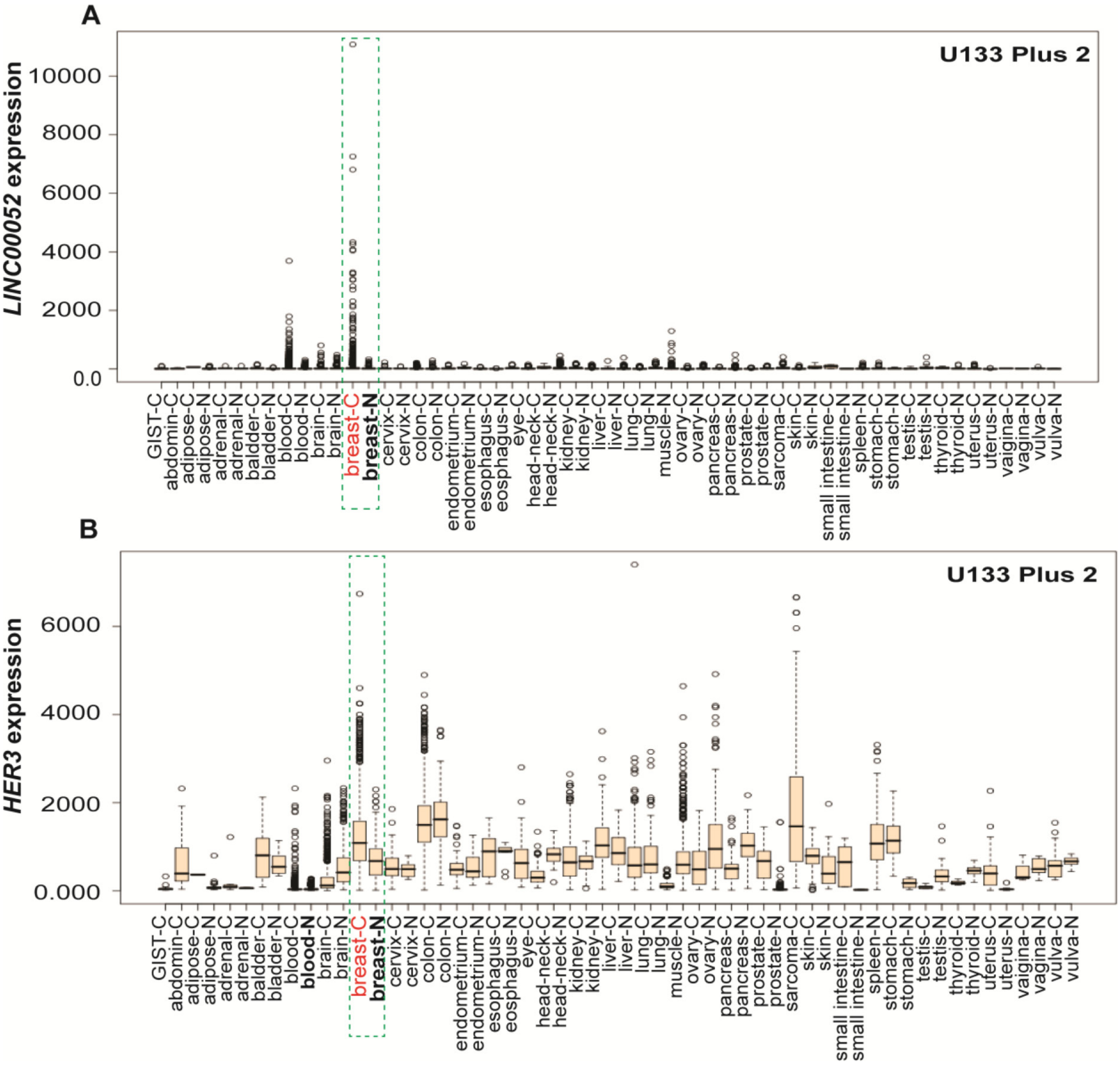

Supplementary Figure S7: (**A**, **B**) *In silico* analysis of *LINC00052* and *HER3* expression. Gene Expression across Normal and Tumor tissue (Gent), http://medicalgenome.kribb.re.kr/GENT/. The database contains a large sample subset data are generated from malignant (indicated as "C") and nonmalignant tissues (indicated as "N").

Supplementary Table S1: Oligonucleotides used in the study

Primer Name	Sequence (5'–3')
LINC0052 cloning	
RV cloning *LINC52*	CGGTGGATCCGAATTGGCCTTGTATAATAACTGGTTTATTTCACTTAACACAACG
RV cloning-FLAG PLVX	CGGTGGATCCGAATTTCACTTGTCATCGTCGTCCTTGTAGTCTGATCCGCCACCTCCACC GGTAGCTGGTCTTGGGGAAGGT
FW cloning LINC52	GGTCGCCACCGAATTACTCAGCTCTCTCACCATGCGATTGCCCTGCAACACC
RV cloning LINC52+U3	CTAGATCCGGTGGATCCAGTTATGGAACAAGAGA
RVcloning LINC52	CGGTGGATCCGAATTGGCCTTGTATAATAACTGGTTTATTTCACTTAACACAACG
FW LINC52_AS_PLVX	GGTCGCCACCGAATTGGCCTTGTATAATAACTGGTTTATTTCACTTAACACAACG
RV LINC52_AS_PLVX	CGGTGGATCCGAATTACTCAGCTCTCTCACCATGCGATTGCCCTGCAACACC
qRT-PCR	
ACTIN FW	CTCTTCAGCCTTCCTTCCT
ACTIN RV	AGCACTGTGTTGGCGTACAG
FW GAPDH	TTGCCATCAATGACCCCTTCA
RV GAPDH	CGCCCCACTTGATTTTGGA
FW1 18S rRNA	GGCCCTGTAATTGGAATGAGTC
RV1 18S rRNA	CCAAGATCCAACTACGAGCTT
FW2 18S rRNA	GTAACCCGTTGAACCCCATT
RV2 18S rRNA	CCATCCAATCGGTAGTAGCG
LINC00052 Fi	GGGAAGATCAGCAAAGCAAAC
LINC00052 Ri	AAGATTTTATTGCCCTAA
LINC00052 Fex3	ATCATAACAATTCATCCTG
LINC00052 Rex3	CGTCACCACAATCAATTT
LINC00052 Rex3.1	AAGATTTTATTGCCCTAA
hEGFR FW1	GGCACTTTTGAAGATCATTTTCTC
hEGFR RV1	CTGTGTTGAGGGCAATGAG
hEGFR FW2	CGAGGGCAAATACAGCTT
hEGFR RV2	AAATTCACCAATACCTATT
HER3/neu FW1	CCTCTGACGTCCATCGTCTC
HER3/neu RV1	CGGATCTTCTGCTGCCGTCG
HER3/neu FW2	CTGAACTGGTGTATGCAGATTGC
HER3/neu RV2	TTCCGAGCGGCCAAGTC
HER2_FW3	GGTCCTGGAAGCCACAAGG
HER2_RV3	GGTTTTCCCACCACATCCTCT
HER3 F1	TGCAGTGGATTCGAGAAGTG
HER3 R1	GGCAAACTTCCCATCGTAGA
HER4 FW	ACAGCAGTACCGAGCCTTTGC
HER4 RV	GCCACTACCACGTAGCCTGTGAC
**FISH-RNA**	
LINC52 Rv ex3+T7	TAATACGACTCACTATAGGGAGAGATGTAGCAAAGCATCACAA
LINC52 Rv ex3+T3	AATTAACCCTCACTAAAGGGAGAGATGTAGCAAAGCATCACAA
